# Viral dsRNA drives hyperinflammatory but blunted antiviral responses with enhanced PD-L1 induction in a COPD-like airway epithelial model

**DOI:** 10.1016/j.bbrep.2026.102710

**Published:** 2026-07-09

**Authors:** Megumi Hayashi, Keiko Ueno-Shuto, Ryunosuke Nakashima, Noriki Takahashi, Tomoki Kishimoto, Mary Ann Suico, Hirofumi Kai, Tsuyoshi Shuto

**Affiliations:** a Department of Molecular Medicine, Graduate School of Pharmaceutical Sciences, Kumamoto University, 5-1 Oe-Honmachi, Chuo-ku, Kumamoto, 862-0973, Japan; b Laboratory of Pharmacology, Division of Life Science, Faculty of Pharmaceutical Sciences, Sojo University, 4-22-1 Ikeda, Nishi-ku, Kumamoto, 860-0082, Japan; c Global Center for Natural Resources Sciences, Faculty of Life Sciences, Kumamoto University, 5-1 Oe-Honmachi, Chuo-ku, Kumamoto, 862-0973, Japan

**Keywords:** COPD, PD-L1, poly(I:C), Airway epithelium, p38 mitogen-activated protein kinase (MAPK), Antiviral immunity

## Abstract

Programmed death-ligand 1 (PD-L1) suppresses T-cell activation during antiviral immune responses, and its expression is increased in airway epithelial cells after viral infection. Because chronic obstructive pulmonary disease (COPD) is characterized by excessive airway inflammation and frequent virus-triggered exacerbations, we investigated the regulation and possible functional relevance of PD-L1 in a COPD-like airway epithelial model in response to viral double-stranded ribonucleic acid (dsRNA) stimulation. Human bronchial epithelial 16HBE14o-cells and β/γ epithelial sodium channel (ENaC)-overexpressing 16HBE14o- (β/γENaC-16HBE14o-) cells were stimulated with polyinosinic:polycytidylic acid [poly(I:C)]. In β/γENaC-16HBE14o-cells, poly(I:C) markedly enhanced the expression of pro-inflammatory mediators, including Toll-like receptor 3 (TLR3), interleukin-6 (IL-6), and tumor necrosis factor-α (TNF-α), along with increased p38 phosphorylation and nuclear accumulation of nuclear factor kappa B (NF-κB) subunits. In contrast, antiviral signaling was attenuated, as shown by reduced interferon regulatory factor 3 (IRF3) phosphorylation and weaker interferon-β (IFN-β) induction compared with parental cells. Notably, poly(I:C)-induced PD-L1 expression was significantly higher in β/γENaC-16HBE14o-cells than in normal airway epithelial cells. Pharmacological inhibition experiments showed that PD-L1 induction depended on p38 signaling rather than NF-κB. In a co-culture system, blockade of programmed cell death-1 (PD-1)/PD-L1 interaction with BMS202 tended to increase IL-2 production by Jurkat T cells exposed to poly(I:C)-stimulated β/γENaC-16HBE14o-cells, with a large effect size. These findings suggest that COPD-like airway epithelial cells display exaggerated inflammatory responses but impaired antiviral responses to viral dsRNA, accompanied by enhanced p38-dependent PD-L1 induction, which may contribute to reduced T-cell activation during COPD exacerbation.

## Highlights

• Poly(I:C) exaggerated inflammatory responses in COPD-like airway epithelial cells.
• COPD-like (β/γENaC-overexpressing) cells showed reduced IRF3 activation and IFN-β induction.
• Poly(I:C)-induced PD-L1 expression was enhanced in a p38-dependent manner.
• PD-1/PD-L1 blockade tended to restore T-cell activation in co-culture.

## Graphical abstract

**Figure:**
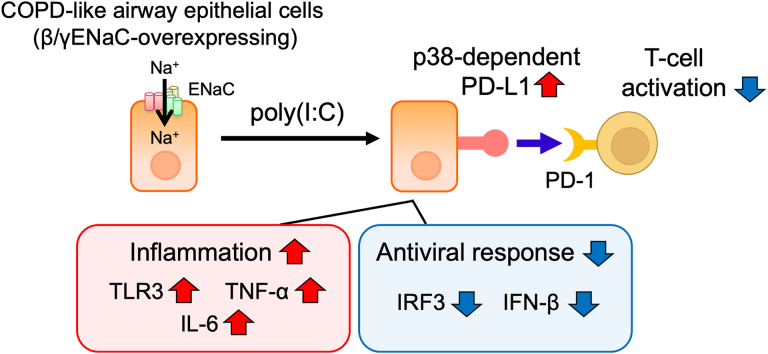

## 1 Introduction

The airway epithelium is a major site of host defense against inhaled respiratory viruses and an active regulator of pulmonary immune homeostasis. Upon sensing viral nucleic acids through pattern recognition receptors such as Toll-like receptor 3 (TLR3), retinoic acid-inducible gene-I (RIG-I), and melanoma differentiation-associated gene 5 (MDA5), airway epithelial cells induce pro-inflammatory mediators and antiviral interferons, thereby coordinating local inflammation, viral restriction, and immune-cell recruitment [[1], [2], [3]]. In chronic airway diseases, however, epithelial barrier dysfunction and innate immune dysregulation can shift this response away from balanced host defense toward persistent inflammation and impaired antiviral protection [3].

Chronic obstructive pulmonary disease (COPD) is characterized by persistent airway inflammation, mucus hypersecretion, and progressive airflow limitation, and respiratory viral infection is a major trigger of acute exacerbations [4,5]. Increasing evidence suggests that the COPD airway epithelium mounts a qualitatively imbalanced response to viral infection rather than a merely quantitative change. In particular, virus-infected COPD airway epithelial cells have been reported to exhibit exaggerated inflammatory signaling, along with deficient or delayed interferon-mediated antiviral responses, a combination that may contribute to a higher viral burden, prolonged illness, and worse exacerbation outcomes [[6], [7], [8], [9]]. These observations raise the possibility that COPD-associated epithelial stress reprograms viral RNA sensing, thereby amplifying inflammation at the expense of effective antiviral immunity.

Programmed cell death-1 (PD-1) and its ligand programmed death-ligand 1 (PD-L1) are immune checkpoint molecules that limit excessive immune activation and shape the magnitude of T-cell responses [[10], [11], [12]]. Although this pathway has been studied extensively in hematopoietic and tumor cells, PD-L1 can also be induced in airway epithelial cells under viral infection or inflammatory conditions [10,11,[13], [14], [15]]. In the respiratory tract, double-stranded ribonucleic acid (dsRNA) stimulation and respiratory viral infection increase epithelial PD-L1 expression and can suppress antiviral T-cell effector function [10,11,[13], [14], [15]]. PD-L1 expression has also been reported to be altered in COPD lungs, suggesting that checkpoint signaling may contribute to immune dysregulation in the COPD airway [12,16]. However, it remains unclear how COPD-like epithelial dysfunction influences PD-L1 regulation during viral infection, particularly in the context of the inflammatory-antiviral imbalance described above.

Among the signaling pathways linking these processes, p38 mitogen-activated protein kinase (MAPK) is of particular interest. p38 is activated by viral infection or viral dsRNA in airway epithelial cells, contributing to the production of inflammatory mediators [[17], [18], [19]]. Moreover, increased p38 activity has been associated with airway inflammation in COPD [19]. At the same time, epithelial sodium channel (ENaC)-driven airway surface liquid depletion and mucus stasis are closely linked to muco-obstructive airway disease and chronic inflammatory remodeling [20,21]. In this context, β/γENaC-overexpressing human bronchial epithelial 16HBE14o-cells provide a useful airway epithelial model with COPD-like features, as they reproduce ENaC-associated epithelial stress relevant to mucus hypersecretory and inflammatory airway disease and have proven informative for dissecting ENaC-dependent epithelial phenotypes [[22], [23], [24], [25]]. This model, therefore, offers an experimentally tractable system to examine how a COPD-like epithelial state reshapes responses to viral dsRNA.

In the present study, we used β/γENaC-overexpressing 16HBE14o-cells and parental 16HBE14o-cells to investigate inflammatory and antiviral signaling, and PD-L1 induction in response to polyinosinic:polycytidylic acid [poly(I:C)], a synthetic analog of viral dsRNA. We further assessed the functional relevance of epithelial PD-L1 using a co-culture system with Jurkat T cells. Our results show that COPD-like airway epithelial cells adopt a hyperinflammatory but antiviral-blunted response to viral dsRNA, accompanied by enhanced p38-dependent PD-L1 induction, and further suggest that PD-1/PD-L1 blockade may partially restore T-cell activation under these conditions.

## 2 Materials and methods

### 2.1 Reagents and antibodies

Poly(I:C) was purchased from InvivoGen (San Diego, CA, USA). SB203580, BAY 11-7082, and BMS202 were purchased from Selleck Chemicals (Houston, TX, USA). Anti-cluster of differentiation 3 (CD3) and anti-CD28 antibodies were obtained from eBioscience (San Diego, CA, USA).

### 2.2 Cell culture and stimulation

Human bronchial epithelial 16HBE14o-cells and β/γENaC-overexpressing 16HBE14o-cells (β/γENaC-16HBE14o-) were cultured on fibronectin-coated dishes (FUJIFILM Wako, Osaka, Japan) in Eagle's minimum essential medium supplemented with 10% fetal bovine serum (FBS), 100 U/mL penicillin, and 100 μg/mL streptomycin [22,23]. Jurkat T cells (ATCC) were maintained in RPMI-1640 supplemented with 10% heat-inactivated FBS, 100 U/mL penicillin, and 100 μg/mL streptomycin. FBS was heat-inactivated at 56°C for 30 minutes (min).

For stimulation experiments, 16HBE14o- and β/γENaC-16HBE14o-cells were treated with poly(I:C) (20 μg/mL) for 3 or 12 h (h). For inhibitor experiments, β/γENaC-16HBE14o-cells were preincubated with SB203580 (10 μM) or BAY 11-7082 (10 μM) for 1 h and then stimulated with poly(I:C) (20 μg/mL) for 3 h. The concentrations of SB203580 and BAY 11-7082 were selected based on previous studies using bronchial epithelial cell models.

### 2.3 Co-culture assay

β/γENaC-16HBE14o-cells (2 × 10^4^ cells/well) were seeded in 24-well plates and cultured for 24 h. Cells were then treated with poly(I:C) (20 μg/mL) for 24 h to allow epithelial responses to develop before co-culture. After pretreatment, the medium was replaced with Roswell Park Memorial Institute 1640 medium (RPMI-1640), and Jurkat T cells (1 × 10^5^ cells/well) were added directly to the epithelial cells. Soluble anti-CD3 (1 μg/mL), soluble anti-CD28 (1 μg/mL), and BMS202 (50 nM) were added at the start of co-culture. After 24 h, culture supernatants were collected for IL-2 measurement. BMS202 was used at a concentration sufficient to inhibit PD-1/PD-L1 interaction.

### 2.4 ELISA

IL-2 concentrations in cell-free supernatants were measured using a Human IL-2 ELISA kit (R&D Systems, Minneapolis, MN, USA) according to the manufacturer's instructions.

### 2.5 RNA isolation and quantitative reverse transcription polymerase chain reaction (RT-PCR)

Total RNA was extracted using RNAiso Plus (TaKaRa, Shiga, Japan). Complementary deoxyribonucleic acid (cDNA) was synthesized from DNase-treated RNA using a PrimeScript RT reagent kit (TaKaRa). Quantitative RT-PCR was performed with SYBR Premix Ex *Taq*II (TaKaRa) on a CFX Connect Real-Time PCR Detection System (Bio-Rad, Hercules, CA, USA) as previously described [22,23]. Relative messenger RNA (mRNA) expression was calculated using the 2^−ΔΔCt^ method with 18S rRNA as the internal control. Primer sequences are listed in Table 1.

**Table 1:** Primer sequences used for qRT-PCR.

Primer	Sequence
hIL-6_QRT-FW	5′-GCACTGGCAGAAAACAACCT-3′
hIL-6_QRT-RV	5′-CAGGGGTGGTTATTGCATCT-3′
hTNF-α_QRT-FW	5′-CAGCCTCTTCTCCTTCCTGA-3′
hTNF-α_QRT-RV	5′-TGAGGTACAGACCCTCTGAT-3′
hIFN-β_QRT-FW	5′-TCCTTGGCCTTCAGGTAATG-3′
hIFN-β_QRT-RV	5′-AGCACTGGCTGGAATGAGAC-3′
hTLR3_QRT-FW	5′-GTTGGCGGCTGGTAATCTTC-3′
hTLR3_QRT-RV	5′-ACCCGATGATCTACCCACAAAC-3′
hMDA5_QRT-FW	5′-TCCAACTGCTGAACCTCCT-3′
hMDA5_QRT-RV	5′-TGCCCATGTTGCTGTTATGT-3′
hPD-L1_QRT-FW	5′-TGGCATTTGCTGAACGCATTT-3′
hPD-L1_QRT-RV	5′-TGCAGCCAGGTCTAATTGTTTT-3′
h18S_QRT-FW	5′-CGGCTACCACATCCAAGGAA-3′
h18S_QRT-RV	5′-GCTGGAATTACCGCGGCT-3′

### 2.6 Protein extraction

Whole-cell lysates were prepared in radioimmunoprecipitation assay (RIPA) buffer containing protease inhibitor cocktail and sodium orthovanadate. Protein concentrations were determined by the bicinchoninic acid assay.

### 2.7 Nuclear extraction

Nuclear extracts were prepared as described previously with minor modifications [26]. Briefly, phosphate-buffered saline (PBS)-washed cell pellets were resuspended in cold hypotonic buffer containing 10 mM 4-(2-hydroxyethyl)-1-piperazineethanesulfonic acid-potassium hydroxide (HEPES-KOH), 10 mM potassium chloride (KCl), 0.1 mM ethylenediaminetetraacetic acid (EDTA), 0.1 mM ethylene glycol tetraacetic acid (EGTA), 1 mM dithiothreitol, and 0.5 mM phenylmethylsulfonyl fluoride (PMSF). After incubation on ice for 15 min, 10% Nonidet P-40 was added and the samples were vortexed briefly. After centrifugation, nuclear pellets were resuspended in extraction buffer containing 20 mM HEPES-KOH, 0.4 M NaCl, 1 mM EDTA, 1 mM EGTA, 1 mM dithiothreitol, and 1 mM PMSF and rotated at 4°C for 15 min. The nuclear lysates were clarified by centrifugation, and protein concentrations were determined by the Bradford assay.

### 2.8 Western blotting

Equal amounts of protein were resolved by sodium dodecyl sulfate-polyacrylamide gel electrophoresis (SDS-PAGE) and transferred to membranes for immunoblotting. The following primary antibodies were used: anti-p50, anti-p65, anti-IRF3, anti-actin, and anti-histone deacetylase 2 (HDAC2) (Santa Cruz Biotechnology, Dallas, TX, USA); anti-phospho-p38 (Thr180/Tyr182), anti-p38, and anti-phospho-interferon regulatory factor 3 (IRF3) (Ser396) (Cell Signaling Technology, Danvers, MA, USA). After incubation with horseradish peroxidase (HRP)-conjugated secondary antibodies, signals were visualized using SuperSignal West Pico chemiluminescent substrate and an LAS-4000 mini imaging system. Band intensities were quantified using ImageJ.

### 2.9 Statistical analysis

Data are presented as mean ± standard error of the mean (S.E.M.). Statistical significance was evaluated by one-way analysis of variance (ANOVA) followed by Tukey-Kramer or Dunnett's multiple-comparison test, as appropriate. Effect size was estimated using Cohen's d. Analyses were performed with GraphPad Prism 10.0 (GraphPad Software, San Diego, CA, USA). The level of significance was set at p < 0.05. In the comparison of co-culture assay, effect size estimates (d) were calculated with d = 0.2 considered as a small effect, d = 0.5 as a medium effect, and d = 0.8 as a large effect.

## 3 Results

### 3.1 Poly(I:C) induces stronger inflammatory responses in β/γENaC-16HBE14o-cells than in 16HBE14o-cells

**Fig. 1:**
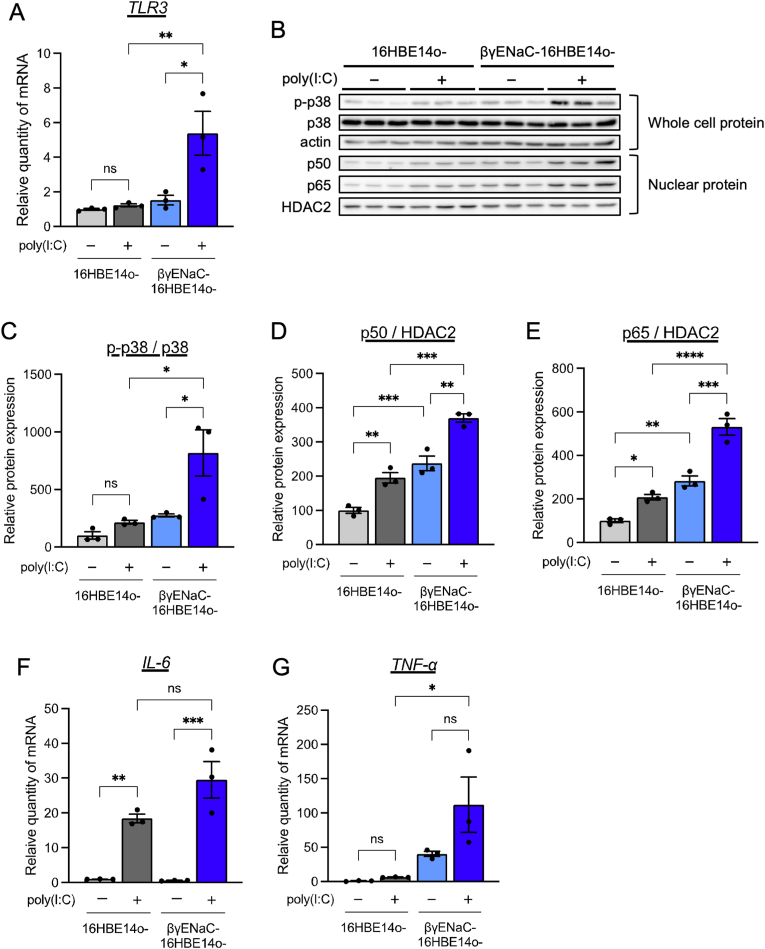
Poly(I:C) induces stronger inflammatory responses in β/γENaC-16HBE14o-cells than in 16HBE14o-cells. (A) 16HBE14o- and β/γENaC-16HBE14o-cells were treated with poly(I:C) (20 μg/mL) for 3 h, and TLR3 mRNA expression was analyzed by qRT-PCR. (B, C) Cells were treated with poly(I:C) for 12 h, and whole-cell lysates were analyzed by western blotting for phospho-p38 and total p38. Actin was used as a loading control. (D, E) Nuclear extracts were prepared after 12 h of poly(I:C) stimulation and analyzed for p50 and p65. HDAC2 was used as a nuclear loading control. Band intensities were quantified using ImageJ. (F, G) IL-6 and TNF-α mRNA expression was analyzed by qRT-PCR after 3 h of poly(I:C) treatment. For qRT-PCR, 18S rRNA was used as the internal control. Data are shown as mean ± S.E.M. (n = 3 wells/group). Statistical analysis was performed by one-way ANOVA with Tukey-Kramer's multiple-comparison test. *P < 0.05, **P < 0.01, ***P < 0.001; n.s., not significant.

To determine how viral dsRNA stimulation affects proinflammatory signaling in airway epithelial cells, 16HBE14o- and β/γENaC-16HBE14o-cells were treated with poly(I:C) and analyzed by quantitative RT-PCR and immunoblotting. Poly(I:C) significantly upregulated TLR3 mRNA in β/γENaC-16HBE14o-cells, whereas no significant increase was observed in parental 16HBE14o-cells (Fig. 1A). Consistent with this finding, poly(I:C) markedly increased p38 phosphorylation in β/γENaC-16HBE14o-cells and produced only a modest, non-significant increase in 16HBE14o-cells (Fig. 1B and C). Because TLR3 signaling activates NF-κB, we next examined nuclear accumulation of the p50 and p65 subunits. Poly(I:C) increased nuclear p50 and p65 in both cell lines, but the increase was more pronounced in β/γENaC-16HBE14o-cells (Fig. 1D and E). In parallel, the proinflammatory cytokines IL-6 and TNF-α were induced by poly(I:C) in both cell lines, with substantially higher induction in β/γENaC-16HBE14o-cells than in parental cells (Fig. 1F and G). These results show that poly(I:C)-induced inflammatory signaling is robustly activated in β/γENaC-16HBE14o-cells compared with parental cells.

### 3.2 Poly(I:C)-induced antiviral signaling is attenuated in β/γENaC-16HBE14o-cells

**Fig. 2:**
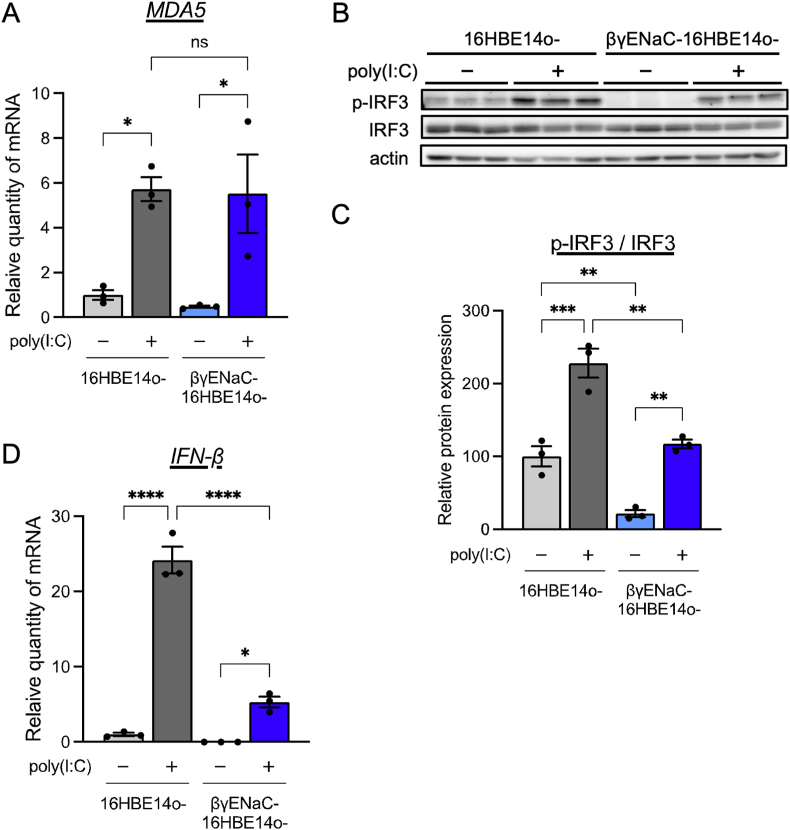
Poly(I:C)-induced antiviral signaling is attenuated in β/γENaC-16HBE14o-cells. (A) 16HBE14o- and β/γENaC-16HBE14o-cells were treated with poly(I:C) (20 μg/mL) for 3 h, and MDA5 mRNA expression was measured by qRT-PCR. (B, C) Cells were treated with poly(I:C) for 12 h, and phospho-IRF3 and total IRF3 were analyzed by western blotting. Actin served as a loading control. Band intensities were quantified using ImageJ. (D) IFN-β mRNA expression was analyzed by qRT-PCR after 3 h of poly(I:C) treatment. For qRT-PCR, 18S rRNA was used as the internal control. Data are shown as mean ± S.E.M. (n = 3 wells/group). Statistical analysis was performed by one-way ANOVA with Tukey-Kramer's multiple-comparison test. *P < 0.05, **P < 0.01, ***P < 0.001; n.s., not significant.

We next examined the antiviral signaling response to poly(I:C). MDA5 mRNA expression was increased to a similar extent in both cell lines after poly(I:C) stimulation (Fig. 2A). In contrast, phosphorylation of IRF3, a key transcription factor downstream of viral RNA sensing, was clearly lower in β/γENaC-16HBE14o-cells than in parental 16HBE14o-cells under both basal and poly(I:C)-stimulated conditions (Fig. 2B and C). Consistent with the reduced IRF3 activation, IFN-β mRNA induction after poly(I:C) stimulation was markedly blunted in β/γENaC-16HBE14o-cells compared with 16HBE14o-cells (Fig. 2D). These findings reveal reduced IRF3 activation and IFN-β induction in β/γENaC-16HBE14o-cells despite MDA5 induction.

### 3.3 Poly(I:C)-induced PD-L1 expression is enhanced in β/γENaC-16HBE14o-cells through p38 signaling

**Fig. 3:**
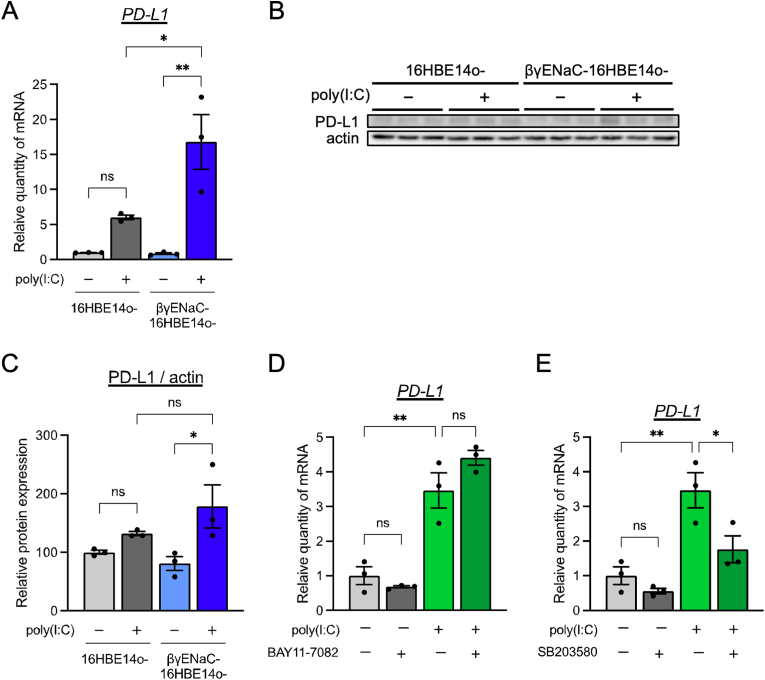
Poly(I:C)-induced PD-L1 expression is enhanced in β/γENaC-16HBE14o-cells and depends on p38 signaling. (A) 16HBE14o- and β/γENaC-16HBE14o-cells were treated with poly(I:C) (20 μg/mL) for 3 h, and PD-L1 mRNA expression was measured by qRT-PCR. (B, C) Cells were treated with poly(I:C) for 12 h, and PD-L1 protein expression was analyzed by western blotting. Actin was used as a loading control. Band intensities were quantified using ImageJ. (D, E) β/γENaC-16HBE14o-cells were pretreated with SB203580 (10 μM) or BAY 11-7082 (10 μM) for 1 h and then stimulated with poly(I:C) (20 μg/mL) for 3 h. PD-L1 mRNA expression was determined by qRT-PCR. For qRT-PCR, 18S rRNA was used as the internal control. Data are shown as mean ± S.E.M. (n = 3 wells/group). Statistical analysis was performed by one-way ANOVA with Tukey-Kramer's multiple-comparison test. *P < 0.05, **P < 0.01, ***P < 0.001.

Because COPD is associated with impaired antiviral T-cell responses, we next focused on PD-1/PD-L1 signaling. Poly(I:C) increased PD-L1 mRNA and protein expression in both cell lines, but the induction was significantly higher in β/γENaC-16HBE14o-cells than in parental 16HBE14o-cells (Fig. 3A–C). These findings suggest that COPD-like epithelial cells respond to viral dsRNA with enhanced upregulation of the immune checkpoint ligand PD-L1. To investigate the signaling pathway responsible for PD-L1 induction, β/γENaC-16HBE14o-cells were pretreated with the NF-κB inhibitor BAY 11-7082 or the p38 inhibitor SB203580 before poly(I:C) stimulation. BAY 11-7082 did not suppress poly(I:C)-induced PD-L1 mRNA expression, whereas SB203580 significantly attenuated the response (Fig. 3D and E). These results suggest that p38 signaling contributes to poly(I:C)-induced PD-L1 expression in COPD-like airway epithelial cells.

### 3.4 PD-1/PD-L1 blockade tends to enhance IL-2 production in co-culture with poly(I:C)-stimulated β/γENaC-16HBE14o-cells

**Fig. 4:**
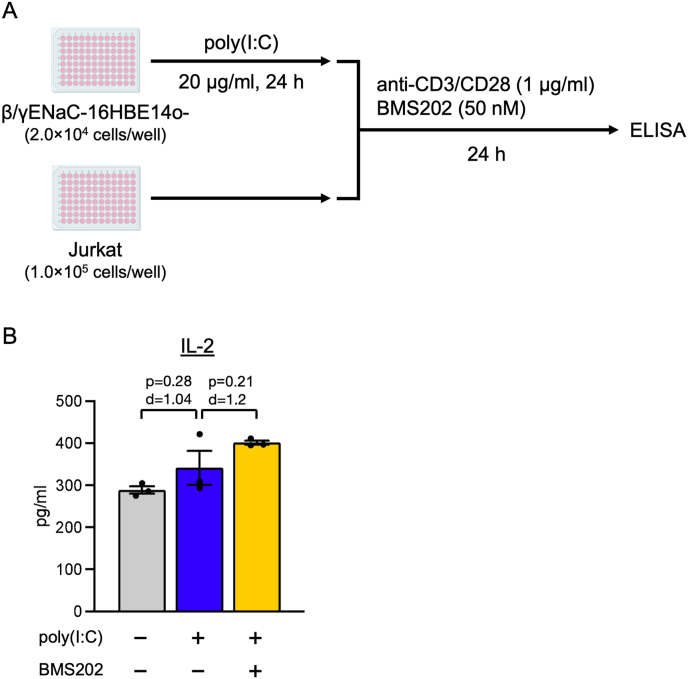
PD-1/PD-L1 blockade tends to enhance IL-2 production by Jurkat cells in co-culture with poly(I:C)-stimulated β/γENaC-16HBE14o-cells. (A) Schematic of the co-culture experiment. β/γENaC-16HBE14o-cells were stimulated with poly(I:C) (20 μg/mL) for 24 h and then co-cultured with Jurkat T cells in the presence or absence of BMS202. (B) IL-2 concentrations in culture supernatants collected after 24 h of co-culture were determined by ELISA. Data are shown as mean ± S.E.M. (n = 3 wells/group). Statistical analysis was performed by one-way ANOVA with Dunnett's multiple-comparison test. Effect size was estimated using Cohen's d.

To examine the possible functional relevance of epithelial PD-L1 induction, β/γENaC-16HBE14o-cells were pretreated with poly(I:C) for 24 h and then co-cultured with Jurkat T cells in the presence or absence of the PD-1/PD-L1 inhibitor BMS202. IL-2 release was measured as an indicator of T-cell activation-associated cytokine production (Fig. 4A). Poly(I:C) pretreatment tended to increase IL-2 production, and BMS202 co-treatment tended to further increase IL-2 levels, with both comparisons showing large effect sizes (d = 1.04 and 1.20, respectively) despite the lack of statistical significance (Fig. 4B). These findings suggest possible involvement of PD-1/PD-L1 signaling in the regulation of T-cell activation-associated IL-2 production in this co-culture setting.

## 4 Discussion

In the present study, we found that β/γENaC-16HBE14o-cells, a COPD-like airway epithelial model, respond to poly(I:C) with a clear imbalance between inflammatory and antiviral signaling. Compared with parental 16HBE14o-cells, β/γENaC-16HBE14o-cells showed higher induction of TLR3, stronger p38 activation, increased nuclear accumulation of NF-κB subunits, and higher expression of pro-inflammatory cytokines, whereas IRF3 activation and IFN-β induction were attenuated. This overall pattern is consistent with previous studies showing that virus-induced COPD exacerbations are associated with exaggerated airway inflammation and impaired, or at least delayed, epithelial antiviral interferon responses [[4], [5], [6], [7]]. Taken together, our findings support the idea that, in a COPD-like epithelial state, viral dsRNA recognition is shifted toward inflammatory amplification rather than balanced host defense.

One possible interpretation of these findings is that chronic muco-obstructive epithelial stress may prime the airway epithelial cells to respond to viral RNA with an inflammatory bias. In βENaC-overexpressing mice, airway surface dehydration alone is sufficient to cause mucus obstruction, persistent neutrophilic inflammation, and emphysema-like changes, supporting the biological plausibility of a pre-inflamed and hyperresponsive epithelial state [20,21]. In addition, previous studies have shown that viral infection or poly(I:C) activates p38 in bronchial epithelial cells, and that p38 contributes to virus-induced production of inflammatory mediators [17,18]. Taken together with our results, these observations suggest that p38 may serve as a signaling hub linking COPD-like epithelial remodeling to enhanced cytokine production. This interpretation is also consistent with human studies of COPD exacerbation, which show that stronger virus-induced inflammation is associated with higher viral burden and worse clinical outcomes [4,5].

A major finding of this study is that poly(I:C)-induced PD-L1 expression was enhanced in β/γENaC-16HBE14o-cells and was suppressed by pharmacological inhibition of p38. These results identify p38 as an important regulator of epithelial PD-L1 induction in a COPD-like setting. Previous studies in airway epithelial cells have shown that dsRNA and rhinovirus induce epithelial PD-L1/B7 homolog 1 (B7–H1) expression and that phosphoinositide 3-kinase δ (PI3Kδ)/NF-κB-, interferon-, and signal transducer and activator of transcription (STAT)-related pathways can modulate this response [[13], [14], [15],^27^]. Our data extend these observations by suggesting that p38 lies upstream of PD-L1 in COPD-like epithelial cells. Notably, PD-L1 induction was enhanced even though IFN-β induction was reduced. This is mechanistically important because type I and type III interferons themselves can increase PD-L1 expression in primary bronchial epithelial cells [15]. Thus, in our model, enhanced PD-L1 cannot be explained simply by stronger antiviral interferon signaling. Rather, the data suggest that COPD-like epithelial stress may uncouple checkpoint induction from canonical IRF3-IFN output, allowing a p38-dominant inflammatory program to maintain or enhance PD-L1 expression even when antiviral competence is reduced [14,15,27]. Because BAY 11-7082 did not suppress poly(I:C)-induced PD-L1 expression under our experimental conditions, NF-κB does not appear to be the dominant pathway controlling PD-L1 induction in this model, although contributions from other pathways cannot be excluded.

This uncoupling may have important functional consequences. Epithelial PD-L1 is not simply a marker of epithelial activation; it can actively suppress antiviral T-cell responses. In a respiratory syncytial virus (RSV)-infected bronchial epithelial/T-cell co-culture model, PD-L1 blockade enhanced CD8^+^ T-cell production of IFN-γ, IL-2, and granzyme B, and reduced viral load [10]. In COPD lung tissue, CD8^+^ T cells exhibit increased PD-1 expression and dysregulated antiviral cytotoxic function, supporting the idea that inhibitory checkpoint signaling contributes to inefficient viral clearance in the chronically inflamed lung [12]. Although the effect of BMS202 in our co-culture system did not reach statistical significance, this limitation should be acknowledged clearly. Even so, the observed trend is consistent with previous studies reporting that epithelial PD-L1 can suppress antiviral T-cell function. More broadly, the increase in epithelial PD-L1 observed here may operate within a wider tolerogenic microenvironment in COPD, since suppressive dendritic-cell pathways and IL-10/IL-27-associated regulatory signals have also been reported in COPD lungs and during rhinovirus-induced exacerbation [28].

Another point worth emphasizing is that PD-L1 dysregulation in COPD is unlikely to be uniform across patients or lung compartments. Recent spatial profiling of human COPD lungs showed that PD-L1 expression can be elevated in bronchioles, parenchyma, vascular wall, and alveolar macrophages in mild-to-moderate disease, whereas expression may be lower in more advanced stages [16]. Therefore, our epithelial model should not be interpreted as evidence that PD-L1 is universally overexpressed in all COPD lungs. Rather, our findings identify a plausible epithelial mechanism by which viral dsRNA exposure may enhance checkpoint signaling under selected COPD-like conditions, particularly when epithelial stress and inflammatory signaling are already increased.

Several limitations should be considered. First, poly(I:C) mimics viral double-stranded RNA but does not reproduce the full complexity of infection with intact respiratory viruses, including viral entry, replication kinetics, and virus-specific immune evasion. Second, although Jurkat cells provide a convenient model for T-cell activation, they do not fully recapitulate the phenotype of primary antiviral CD8^+^ T cells in COPD. Third, because our analysis focused on epithelial PD-L1 regulation, we did not examine the potential contributions of PD-L2, macrophage- or dendritic-cell checkpoint pathways, or soluble immunoregulatory mediators. Future studies using primary COPD airway epithelial cells differentiated at an air-liquid interface, together with authentic rhinovirus or RSV infection and primary human T cells, will be needed to determine whether p38 inhibition can simultaneously reduce epithelial PD-L1 expression and inflammatory cytokine production while preserving, or even restoring, antiviral IRF3/interferon responses. Fourth, PD-L1 expression was not directly quantified at the exact 24-h poly(I:C) pretreatment time point used for the co-culture assay in β/γENaC-16HBE14o-cells. Therefore, the co-culture results should be interpreted as being consistent with a role for epithelial PD-L1/PD-1 signaling, rather than as direct evidence that PD-L1 was increased at the same time point used for T-cell co-culture.

In summary, COPD-like airway epithelial cells exhibit exaggerated inflammatory signaling, attenuated antiviral signaling, and enhanced p38-dependent PD-L1 induction in response to viral dsRNA. These findings suggest that epithelial PD-L1 may contribute to impaired antiviral immune regulation during COPD exacerbation and identify p38-linked PD-L1 induction as a potential mechanistic axis linking epithelial stress, hyperinflammation, and checkpoint-mediated immune restraint in COPD [4,7,12].

## Declaration of generative AI and AI-assisted technologies in the manuscript preparation process

During the preparation of this work, the authors used ChatGPT (GPT-5.4) to improve language and readability. After using this tool, the authors reviewed and edited the content as needed and take full responsibility for the content of the published article.

## Funding

This work was supported by Japan Society for the Promotion of Science (JSPS) KAKENHI JP23K06150 (to T.S.), the Health Life Science S-HIGO Professional Fellowship Program, the Program for Fostering Innovators to Lead a Better Co-being Society JPMJSP2127; Ministry of Education, Culture, Sports, Science and Technology (MEXT), Japan, and Nagai Memorial Research Scholarship from the Pharmaceutical Society of Japan (N-197203 to R.N. and N-217201 to N.T.).

## CRediT authorship contribution statement

**Megumi Hayashi:** Conceptualization, Formal analysis, Investigation, Writing – original draft. **Keiko Ueno-Shuto:** Formal analysis, Investigation, Writing – original draft. **Ryunosuke Nakashima:** Conceptualization, Formal analysis, Funding acquisition, Investigation. **Noriki Takahashi:** Funding acquisition, Investigation. **Tomoki Kishimoto:** Investigation. **Mary Ann Suico:** Writing – review & editing. **Hirofumi Kai:** Conceptualization, Funding acquisition, Supervision. **Tsuyoshi Shuto:** Conceptualization, Formal analysis, Funding acquisition, Investigation, Supervision, Writing – review & editing.

## Declaration of competing interest

The authors declare that they have no known competing financial interests or personal relationships that could have appeared to influence the work reported in this paper.

## Data Availability

The data that support the findings of this study are available from the corresponding author upon reasonable request.
