# Supplementary material for: Viral dsRNA drives hyperinflammatory but blunted antiviral responses with enhanced PD-L1 induction in a COPD-like airway epithelial model
Source: Biochem Biophys Rep. 2026 Jul 9;47:102710. doi: 10.1016/j.bbrep.2026.102710 (PMC13380008; doi:10.1016/j.bbrep.2026.102710)

Viral dsRNA Drives Hyperinflammatory but Blunted Antiviral Responses with Enhanced PD-L1 Induction in a COPD-Like Airway Epithelial Model

Megumi Hayashi, Keiko Ueno-Shuto, Ryunosuke Nakashima, Noriki Takahashi, Tomoki Kishimoto, Mary Ann Suico, Hirofumi Kai, and Tsuyoshi Shuto

Source Data
uncropped blots

Source data of Figure 1B: uncropped blots

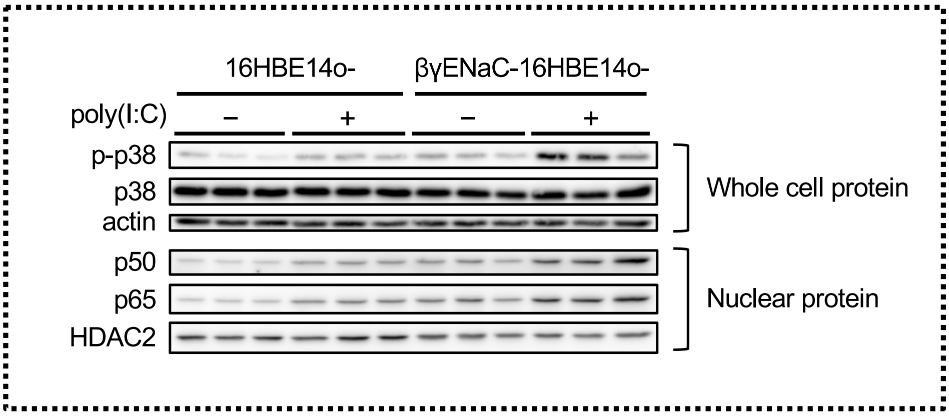

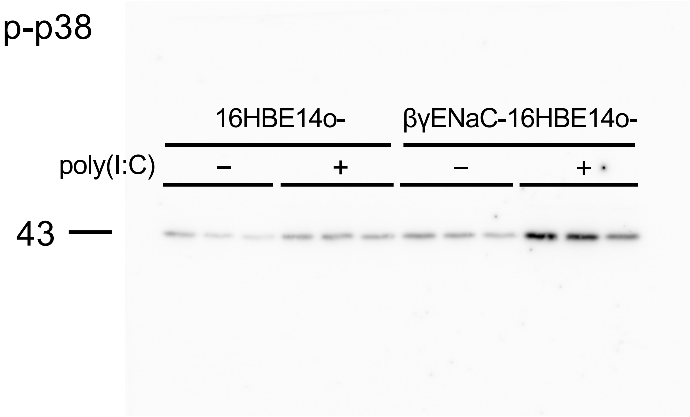

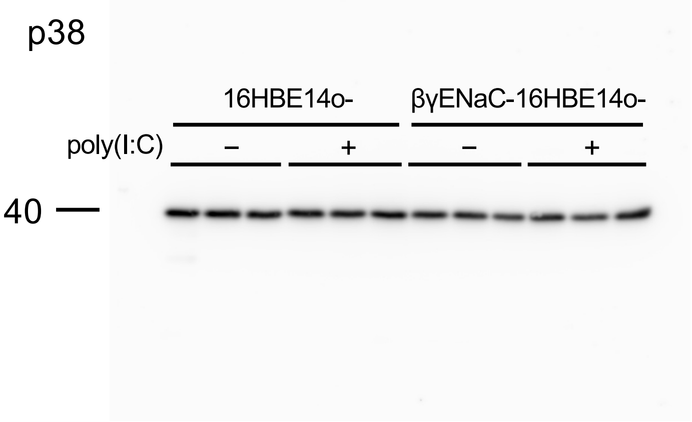

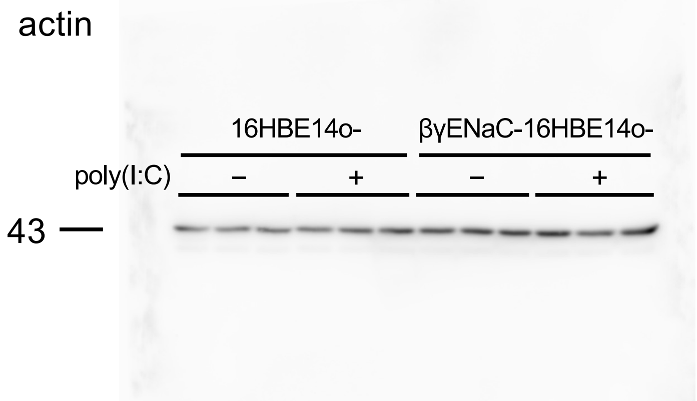

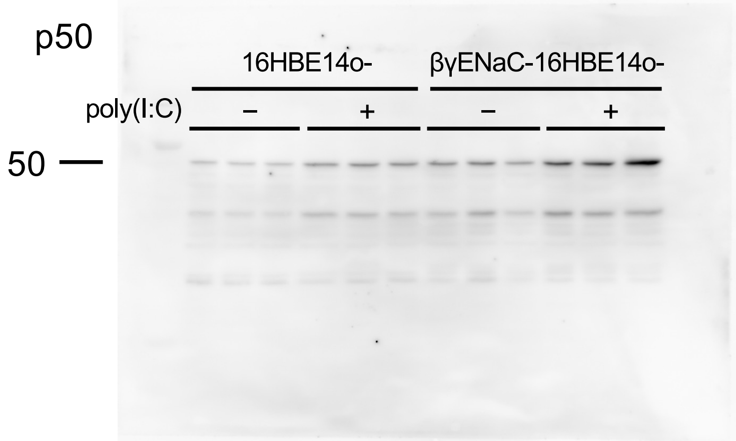

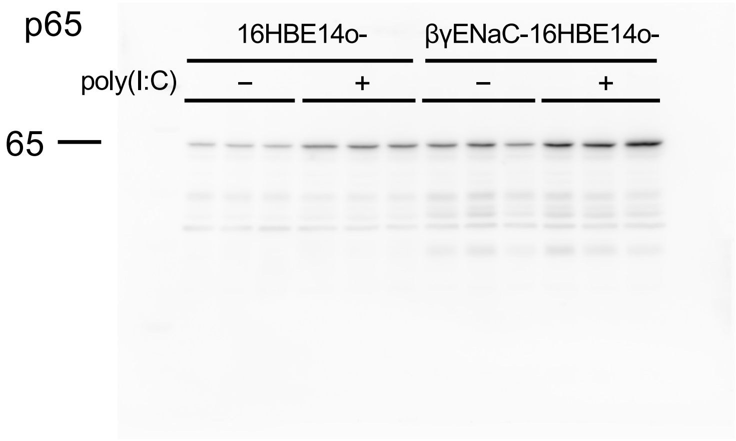

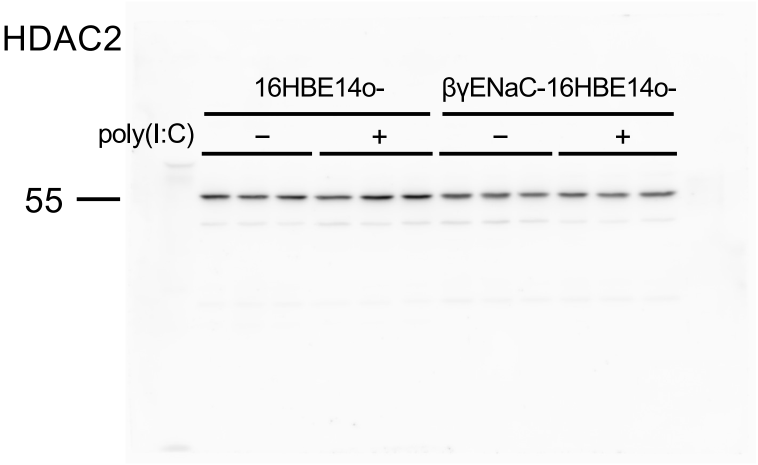

Source data of Figure 2B: uncropped blots

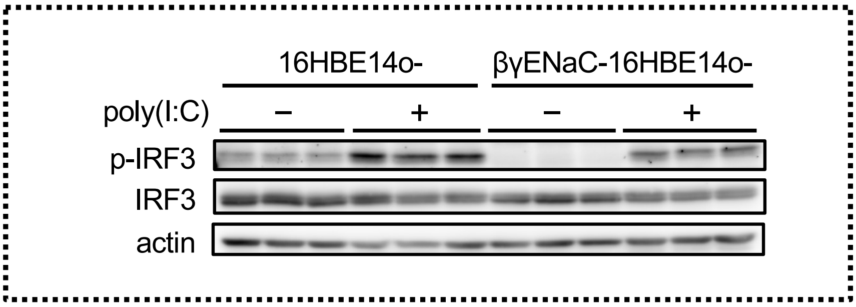

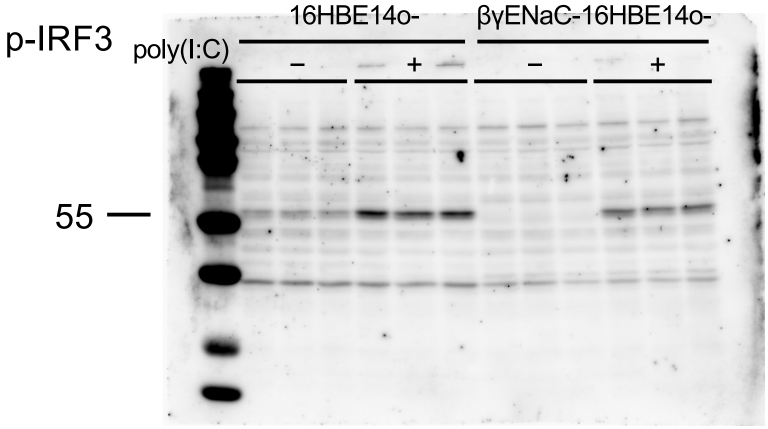

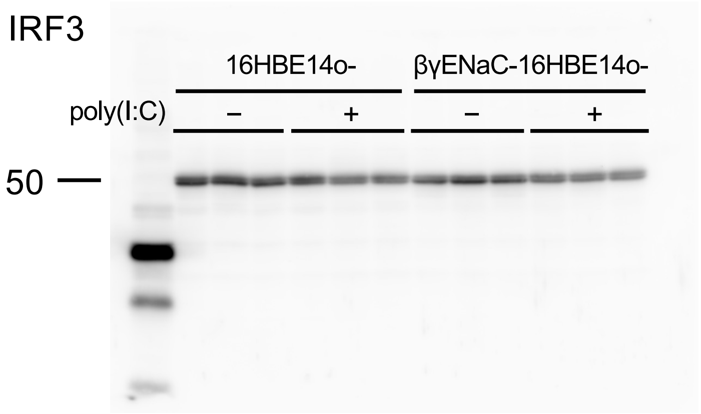

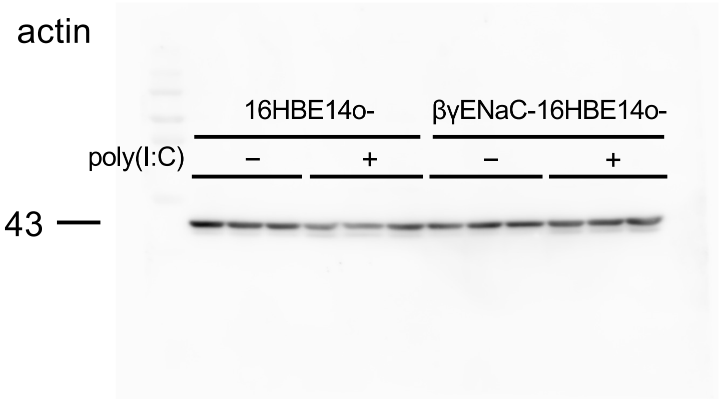

Source data of Figure 3B: uncropped blots

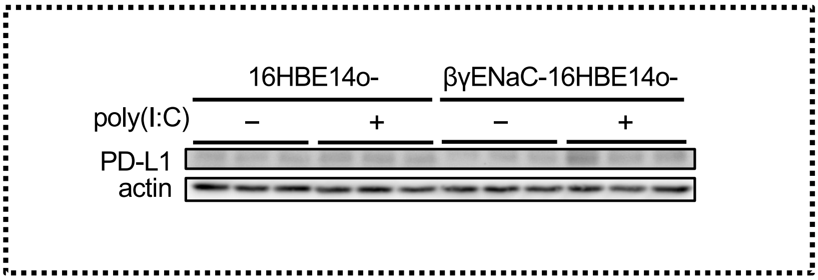

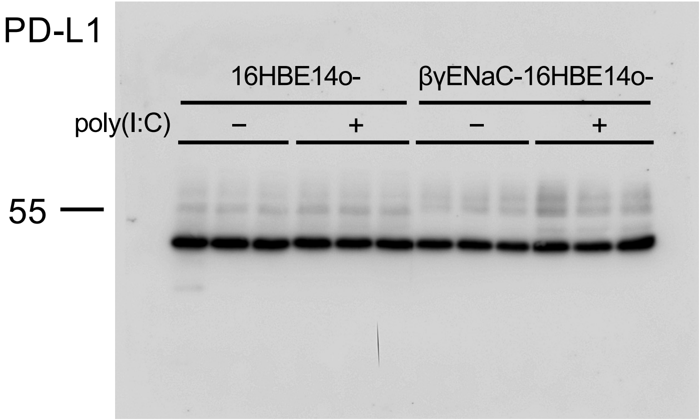

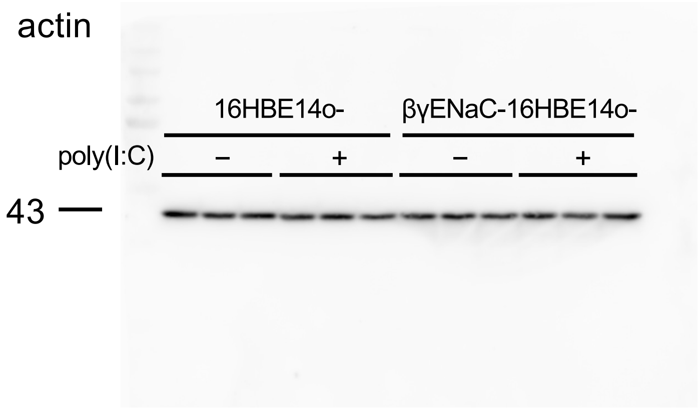

Supplement: Multimedia component 1 [file mmc1.pdf]
